# Rapid detection of pesticide in milk, cereal and cereal based food and fruit juices using paper strip-based sensor

**DOI:** 10.1038/s41598-021-96999-w

**Published:** 2021-09-22

**Authors:** Vaishali Dasriya, Ritu Joshi, Soniya Ranveer, Vishal Dhundale, Naresh Kumar, H. V. Raghu

**Affiliations:** 1 Microbial Biosensors, Food Safety and Quality Assurance Lab, Dairy Microbiology Division, National Dairy Research Institute, Karnal, Haryana India; 2 National Referral Centre, Dairy Microbiology Division, ICAR-National Dairy Research Institute, Karnal, Haryana 132001 India

**Keywords:** Biological techniques, Environmental sciences, Natural hazards

## Abstract

The study was aimed to validate paper strip sensors for the detection of pesticide residues in milk, cereal-based food, and fruit juices in comparison with GC–MS/MS under field conditions. The detection limit of pesticide using rapid paper strip sensor for organophosphate, carbamate, organochlorine, fungicide, and herbicide group ranges from 1 to 10, 1–50, 250–500, 1–50, and 1 ppb, respectively in milk and milk product, cereal-based food and fruit juices. Among 125 samples of milk samples collected from the market 33 milk samples comprising 31 raw milk and 2 pasteurized milk found positive for pesticide using the strip-based sensor. In cereal based food and fruit juice samples, 6 cereal flours and 4 fruit juices were found positive for pesticide residues. The pesticide positive samples were further evaluated quantitatively using GC–MS/MS wherein 7 samples comprised of raw milk, pasteurized milk, rice flour, wheat flour, maize flour, apple juice, and pomegranate juice have shown the presence of chlorpyrifos, chlorpyrifos-methyl, α-endosulfan, β-endosulfan DDD and DDT at trace level as well as at above MRL level. It is envisaged that the developed paper strip sensor can be a potential tool in the rapid and cost-effective screening of a large number of food samples for pesticide residues.

## Introduction

Pesticides are extensively used to shield agricultural produces from pests and weeds. The widespread of pesticides has created serious problems regarding the effect on ecosystems and concern for human health. Pesticides are known to be a potential carcinogen and their effect on humans and their occurrence in diverse foods including dairy products and cereal-based food. Pesticide deposits in food items have been related to a wide assortment of human illnesses, extending from short to long-term harmful effects with toxigenic impact^1,2^. Due to high human health risks, there is a need to constantly monitoring the pesticide exposure or content in the food. In the total top, 10 brands of processed food made up of cereals were contaminated with different 14 organochlorine pesticide which having the nutritional property, nourishes our body and it is the basic level of food which consume in daily diet. It contributes 60% of total worldwide crops. But it is contaminated with pesticides which contribute to high health risks for infants and young children. According to the Akoto et al.^3^ evaluation of these pesticides was found above MRLs which raises worries of conceivable cancer-causing nature of pesticide for infant and youngsters. Pesticide utilization constrained by various national and global administrative bodies. In India, the pesticide guidelines are classified by the Central Insecticides Board and Registration Committee (CIBRC) and the Food Safety and Standard Authority of India (FSSAI). FSSAI has given MRLs of various pesticide groups for different food categories including milk, cereals, and fruit juices. Monitoring the presence of the toxigenic pesticide in food commodities needs a tall affectability and precision since these chemicals are frequently found at minute levels^4^. These are normally being observed quantitatively as well qualitatively by traditional strategies like chromatography-mass spectrometry (LC–MS), gas chromatography-mass spectrometry (GC–MS), and by other spectroscopic techniques^5^. While, traditionally used techniques are reliable, effective, and delicate, but it has also disadvantage like a sophisticated instrument, difficult to handle require a trained technician and also having complex extraction protocol and can't be utilized under field conditions. Consequently, the research concentrated on discovering quick and sensitive devices. Biosensors are being used to explicit sensitive and specific recognition of interested pesticide by using complex combinations of a cellular constituent of microorganisms, spores-based enzyme, chemicals, antibodies, DNA sequences, aptamers, or other compounds. Since biosensors performed as quantitative detection techniques depend on the enzyme-based inhibition in the presence of pesticides^6^.

Spore-based biosensors are cost-effective, easy to use, portable, less time-consuming, simple, robust, and reasonable techniques^7^. Bacterial spore act as a biosensing element for detecting aflatoxin M1 and antibiotic residues in milk is based on the principle that the release of Dipicolinic acid during germination of spores based as a signal^8–10^. In this way, the methodology was additionally extended out to target pathogens, for example, *Enterococci, L. monocytogenes*, and *E. coli* in milk^11–13^.

Pesticide is the toxic chemical can inhibit the acetyl cholinesterase enzyme system in eukaryotic cells that degrade neurotransmitter acetylcholine into acetate and choline in central nervous system of human, pest and rodents. Over exposure of pesticide cause increase in concentration of acetylcholine in brain and blood which has harmful effect to host^14^. IN case of prokaryotic microorganisms like *Bacillus megaterium* are associated with the cell bound esterase^15^. Germination of spore releases various biomarker like enzyme, dipicolinic acid, divalent cation, ATP and nucleic acid^16^. In the current project, we have targeted esterase enzyme in bacterial spores as a target marker enzyme that get inhibited in the presence of pesticide residues. These marker enzyme cab hydrolyses specific chromogenic substrate like Indoxyl acetate and releases blue color chromogenic. While in the absence of pesticide residues in the environment this activity get inhibited so there is no hydrolysis of indoxyl acetate lead to release of blue colored compound. Strip-based innovation for targeting pesticides in milk, cereal, and fruit juice by utilizing novel marker catalysts from prokaryotic sources has been created. The spore enzyme-based sensor is based on the principle of *B. megaterium* spore germination-enzyme inhibition in the presence of pesticide followed by the reaction with chromogenic substrate indoxyl acetate and detection using the paper strip by presence or absence of pesticide through a color change, showing semi-quantitative detection of the target analyte. In the presence of pesticide residues inhibit the esterase enzyme released during germination of spore into vegetative cell and enzyme is unable to convert indoxyl acetate into colorful product i.e., indigo. Thus, the presence of pesticide in the product showing the colorless strip in contrast to the absence of pesticide show change in color in strip to blue color. In the developed spore enzyme based sensor working on the principle of *Bacillus megaterium* spore germination-enzyme inhibition in the presence of pesticide residues followed by the reaction with chromogenic substrate i.e. indoxyl acetate with no color change on paper strip sensor while in the absence of pesticide residues in the sample, there was a release of marker enzyme and hydrolysis of substrate in to blue color compounds with visible color change of paper strip sensor from colorless to blue color. Further, strip-based sensor is very compatible with another chemical, easily available at low cost, passive transport of liquid-like pesticide and organic solvent, versatile in nature with fast response. It is easy to show the result of the presence and absence of pesticides by a change in color.

Acetylcholine esterases represent a various group of hydrolases catalyzing cleavage and formation of ester bonds and are also known as ester hydrolases^17^^.^ These are broadly distributed in animals, flora, and microorganisms^18^. Membrane sure localization of AChE had been mentioned in many microbes. The presence of mobile esterases in *B. megaterium* 20–1 was found by Jung et al.^19^. Colorimetric esterase assay normally makes using chromogenic substrates like indoxyl acetate^20,21^. This principle was applied for the milk and milk products, cereals, and fruit juices by extracting pesticide and detecting it with the paper strip-based sensor. Keeping in perspective on current guidelines and the presence of disease-causing pesticides in our basal diet, there is a need for a fast and sensitive method for detecting pesticides in milk and cereal-based food as well as fruit juices.

## Material and method

### Media and their composition

All media ingredients for preparation of nutrient agar, tryptone glucose yeast extract, and sporulation media are from Himedia. Tryptone glucose yeast extract (pH = 7.0) prepares by addition of tryptone (5.0 g/L), dextrose (2.5 g/L), and yeast extract (1.0 g/L). Preparation of sporulation media require Beef extract (0.074 g/L), Peptone (0.374 g/L), Sodium chloride (0.374 g/L), and yeast extract (0.150 g/L).

### Reagents

Propagation (TGYE) medium was obtained by dissolving tryptone (0.5%), dextrose (0.1%), and Yeast extract type-1 (0.25%) in distilled water. The pH of the medium was adjusted to 7.0 followed by sterilization at 121 °C for 40 min. Store it at room temperature. The chromogenic substrate was prepared in 1 mL organic solvent with addition of 3.5 mg indoxyl acetate (chromogenic substrate) (Sigma Aldrich, U.S.A; Hi-Media, Mumbai, India). Pesticide (Supleco, Sigma-Aldrich, U.S.A.) solution prepared by diluting standard stock solution in acetonitrile (Fisher Scientific, HPLC Grade, U.K.) and stored at 4 °C. Potassium Phosphate Buffer (Hi-Media, India) (pH 6.8) was prepared by dissolving dipotassium hydrogen phosphate (K_2_HPO_4_) (0.174 g) and potassium dihydrogen phosphate (KH_2_PO_4_) (0.136 g) in 100.0 mL of distilled water and stored at room temperature.

### Instrumentation

Inoculation and reconstitution of spore carried out in Bio-safety Level-II cabinet (Esco Biotech Pvt. Ltd., India). Incubator shaker (Eppendorf, Inc., USA.) was used for the incubation of spore. Separation of pellet done in centrifuge (Eppendorf, U.S.A.). Spore stored in − 20 °C Deep Freeze (Bluestar, India). Lyophilization of spores were carried out in Lyophilizer (Labconco, U.S.A.). Microbiological plate reader (Perkin Elmer, U.S.A.) used for adjustment of spore O.D. Immobilization of substrate on paper strip carried out using the Easy printer (Advanced sensor system Pvt. Ltd., India). Usually block heater (Labnet International, Inc., U.S.A) used for evaporation of organic solvent from extracted pesticide solvent of food sample.

### Production of *B. megaterium* MTCC 2949 spore

Production of *B. megaterium* MTCC 2949 spore was carried out as per the protocol explained by Kumar et al.^9^.

### Preparation of functionalized paper strip

Cut the paper strips in dimensions of 0.5 × 3.5 cm. immobilized the chromogenic substrate indoxyl acetate using an easy printer. Approximately 400 µL substrate dispense to needle and loaded substrate using printer needle on the paper. Developed functionalized paper strip vacuum-packed INDVAC vacuum packaging machine.

### Lyophilisation of spores

Dispense 20–40 μL of final spore (OD 0.32 ± 0.02) and reconstitute it with 30 μL phosphate buffer (10 mM). Add functionalized paper strip followed by incubation at 37 °C for 10–20 min. Observed blue color for a recording test time of enzyme activity at the time interval between the incubation periods before the lyophilization. Lyophilized 20 μL of spore to lyophilizer at − 84 ± 1 °C under vacuum of 1 ± 0.5 torr (1 Torr = 133.33 Pascal) for 1 h. After finishing it packed within a plastic bag and stored at − 20 °C and 4 °C.

### Extraction of pesticide from cereal based foods

Take equal volume (500 µL) sample and acetonitrile was taken in a micro-centrifuge tube and vortex it for 1 min. Centrifugation was done at 10,000 rpm for 5 min at 37 °C. The supernatant (~ 750 μL) was separated in to tubes containing 0.25 g sucrose (reagent 1) (Hi-Media, Mumbai, India) and subsequently, vortex for 1 min. Centrifugation was done at 10,000 rpm for 5 min at 37 °C. Then separate, a top layer containing solvent ~ 400 μL and collected it into a tube containing 0.25 g PSA (reagent 2) (Supleco, Sigma-Aldrich, U.S.A.) and MgSO_4_ (Hi-Media, Mumbai, India)) at ratio of 1:2 and vortex it properly followed by centrifugation at 10,000 rpm for 5 min at 37 °C. Top layer containing solvent ~ 200 μL was separated and filtered through specialized filter tips. The filtrate was collected in a micro-centrifuge tube and allowed to evaporate using a dry block heater at 80 °C for 15–20 min. Finally, the tube containing pesticide residue (Tube-2) was used to carry out paper strip assay^16^.

### Assay protocol for pesticide detection using a paper strip

Transfer reconstituted spore with 30 μL of potassium phosphate buffer (pH 6.8) to tube containing residues left after evaporation of ~ 250 μL and mixed by vortexing for 1 min. After mixing it properly, tubes were allowed to incubate at 37 °C for a period of 40 min (exposure time). After exposure, the mixture was vortexes for 25 s. and the paper strip was added and followed by incubation at 37 °C for 10–15 min. During incubation, the tubes were observed for the colour development on the paper strip. Development of blue colour indicates an absence of pesticide residues and the presence of pesticide residues was indicated by less or no blue colour development. For the comparison of results, positive control (Paraxon methyl), negative fresh samples were screened by Acetate QuEChERS method.

### Protocol for the limit of detection (LOD) of pesticide from food products

Initially, the different concentrations of pesticide prepared in organic solvent i.e., acetonitrile, and followed the protocol of pesticide detection. Similarly, a homogenous mixture of products spiked with different quantities of various pesticides. The spiked concentration of pesticide from 100 ppm to 1 ppb. Spiked product proceeds for extraction of pesticide and then follows assay protocol for pesticide detection using a paper strip. The minimum concentration at which the marker enzyme activity get inhibited with no colour change (remains colourless) on paper strip based sensor and it was indicated as a LOD of the paper strip based sensor as compared with positive control and negative control. Further, different food products samples namely milk, cereal based foods, fruits and fruit juices were screened for pesticide residues using paper strip based sensor. The positive samples among them were further analysed by specific and quantitative detection using GC–MS/MS method.

### GC–MS/MS analysis

#### Sample preparation

Equal quantity of homogeneous food sample and acetonitrile transfer in a 50 mL polypropylene tube. Kept this mixture for 5 min at room temperature followed by the addition of 6.0 g of MgSO_4_ and 1.5 g of sodium acetate followed by vertex for proper mixing and centrifugation at 6000 rpm for 10 °C. Then, collect 1.5 mL of supernatant and transferred it to dispersive solid-phase extraction tubes (d-SPE). Afterward vortex it properly for 1 min and centrifuge was carried out at 6000 rpm at 10 °C for 5.0 min. approximately, 1 mL of the clear extract was injected into GC for analysis.

#### Conditions for GC–MS/MS

Estimation of pesticide performed by GC–MS/MS composed with SLB-5MS, (30 m × 0.25 mm × 0.25 μm, Supelco, Sigma Aldrich) and TQ 8030 triple quadrupole detector. The initial temperature of the GC oven was 80 °C for 2 min and raised at 20 °C /min up to 180 °C with no holding period and further, it was raised for 5 °C /min up to 300 °C for 3 min. Electron impact mode was used for performing mass spectrometry and ionization energy was 70 eV with solvent delay time 3 min. The quadrupole detector voltage was 0.6 kV. The injection temperature was 250 °C and the carrier gas used was helium.

### Statistical analysis

Pesticide detection data generated through paper strip sensor and GC–MS/MS analysis were evaluated for precision, accuracy, correlation coefficient, limit of detection, limit of quantification, repeatability, reproducibility based on the method given by NATA^22^ (2012). All the experiments were carried out in triplicate (n = 3).

## Results and discussion

### LOD of paper strip for pesticide in spiked food sample

**Figure 1:**
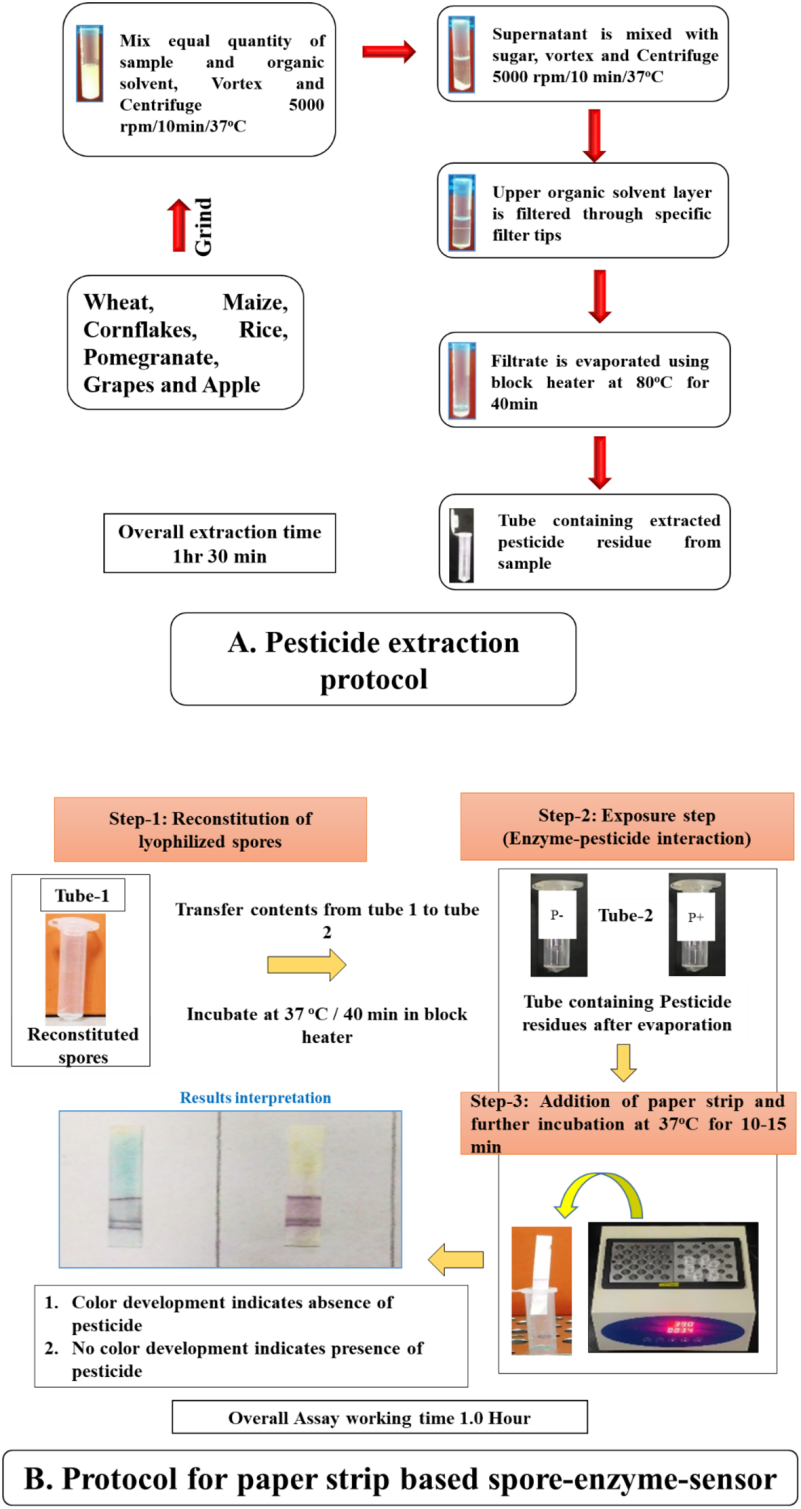
Paper strip assay for rapid detection of pesticide residues in Food. (**A**) Protocol for extraction and screening of Pesticide residues in food products. (**B**) Color of Paper strip before (Colorless) and after (Blue) incubation in the detection of pesticide residues. (+ ve) sample—Colorless, (− ve) sample blue color.

In the present study, different groups of pesticide concentrations are prepared in organic solvent and detected using the paper strip assay (Fig. 1A). In the presence of pesticide residues like organophosphate, carbamate, organochlorine, and fungicide and herbicide group, the bacillus spores present in the tubes were germinated into vegetative cells followed by the release of marker enzyme, and activity of the marker enzyme get inhibited due to the presence of pesticides residues present in the food system. Therefore, there was no colour change due to no hydrolysis of a specific chromogenic substrate, and the strip remains colourless. In the absence of pesticide residues in food, marker enzyme released during spore germination will be intact and hydrolyze the specific chromogenic substrate with the production of blue colour (Fig. 1B).

The developed paper strip assay was evacuated with a different group of pesticides including organophosphate, carbamate, organochlorine, and fungicide and herbicide group spiked in a pure system as well as a different food system in the range from 100 ppm to 1 ppb in an organic solvent for the detection of Limit of Detection (LOD) of the developed assay. The concentration on which paper strip showing no colour change i.e., LOD of the paper strip based sensor (the concentration of pesticide detected by paper strip based sensor. The limit of detection in a pure solvent system ranges from 1 to 10 ppb, 1–50 ppb, 250–500 ppb, 1–50 ppb, and 1 ppb for organophosphate, carbamate, organochlorine, and fungicide, and herbicide, respectively (Table 1). Further, different concentrations of pesticides spiked inhomogeneous food samples like milk, cereal-based food, and fruit juice sample. After extraction of pesticide, the pesticide residues analysed by paper strip assay protocol and the LOD of an organophosphate pesticide, carbamate pesticide, organochlorine pesticide, fungicide, and herbicide group in milk ranges from 1–10 ppb, 1–50 ppb, 250–500 ppb, 1–50 ppb, and 1 ppb, respectively by strip-based sensor same as obtained in pure system. The LOD for cereal-based food was found to be in the range of 1–100 ppb, 10 ppb, 1 ppb, and 1–100 ppb for organophosphate, organochlorine, fungicide, pyrethroid ester, and herbicide, respectively. The LOD for fruit juices are in the range from 1 ppb, 1–10 ppb, and 1 ppb, respectively for organophosphate, fungicide, and herbicide groups. There was no difference in a detection limit of pesticide in the pure system and food sample was observed using paper strips.

**Table 1:** LOD of different group of pesticide from cereal based foods sample.

Pesticides	Regulatory MRL limits (ppb)	Limit of detections (ppb) in spiked food samples	Limit of detection (ppb) in pure system
**Insecticides—organophosphate group**			
Primiphos methyl^b^	500	10	10
Triazophos^a^	50	1	1
Anilophos^a^	100	100	100
**Insecticides—organochlorine group**			
Lindane^c^	10	10	10
Fungicide			
Iprodione^a^	10,000	1	1
Propineb^a^	50	1	1
**Pyrethroid ester**			
Deltametrin^b^	10	1	1
**Herbicide**			
Diuron^a^	500	10	10
Mesosulfuron methyl^b^	10	1	1
Metribuzin^b^	30	10	10
Pendimethalin^a^	50	10	10
Penoxsulum^a^	100	100	100
Pyrazosulfuron ethyl^a^	10	10	10
Pretilachlor^c^	50	10	10
Propiconazole^c^	10	10	10

^a^Rice, ^b^Wheat, ^c^Maize.

### Screening of food sample using paper strip assay

#### Cereal based foods

**Figure 2:**
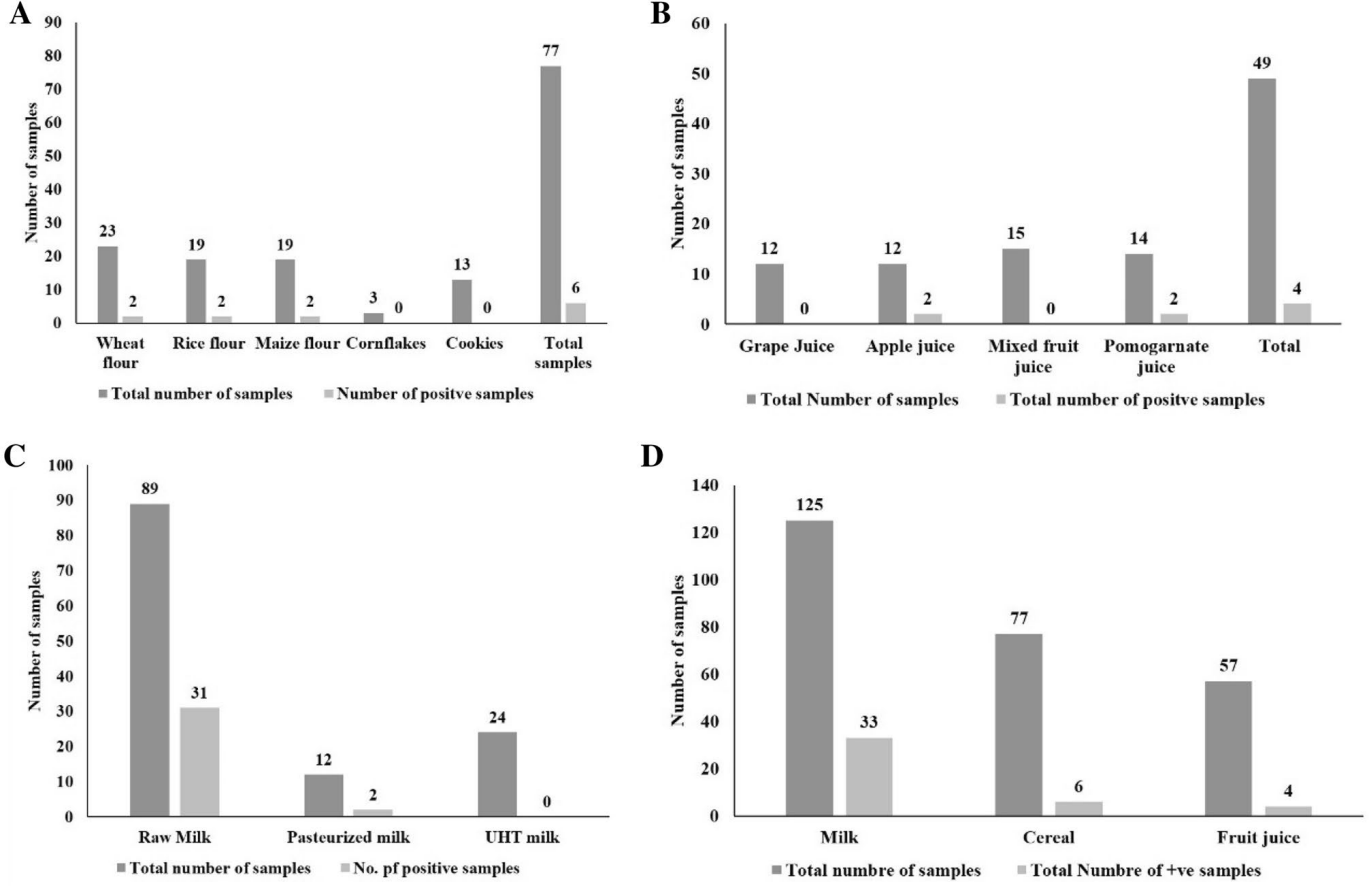
Screening of food sample using paper strip assay. (**A**) Incidence of pesticide residues in cereal based products. (**B**) Incidence of pesticide residues in Fruit juices. (**C**) Incidence of pesticide residues in milk samples. (**D**) Overall incidence of pesticide residues.

Cereal-based foods, fruit juices, and milk were screened for pesticide detection. Cereal-based food comprises a total of 19 samples of rice flour, 23 samples of wheat flour, 12 samples of maize flour, 3 samples of cornflakes, and 13 samples of cookies were screened. Among these, 3 each sample of wheat flour, rice flour, and maize flour samples have shown the presence of pesticide residues by not changing the colour from colorless to blue colour (Fig. 2A). There were no positive samples found for corn flakes and cookies. While the using modified QuEChERS and GC–MS were used for the detection of pesticide in whole wheat flour samples collected from the south Brazil Region^23^. The detection of fenitrotion in 20% of maize flour samples found less than permissible limits^24^. The results indicate that different flour and products need continuous checking to warrant food safety.

#### Fruit juices

In the case of fruit juices, a total of 57 samples of fruit juice collected from the market comprises 12 grapes juice samples, 15 apple juice samples, 15 mixed fruit samples, and 14 pomegranate juice samples. All sample screens for pesticide detection using the paper strip-based sensors. Among the 57 samples, 2 raw un-washed pomegranates and 2 raw un-washed apple juice found positive for pesticide at their MRL level (Fig. 2B). However, these pesticides are harmful and not deteriorate naturally and they will present on plant tissue and appear in the pulp and juice. However, the pesticide cannot completely remove from the pulp and juice. Since wherein natural fruit juice has less concentration of pesticide than the processed juice. Children consume more juice than adults and thus the children are more susceptible than adults^25^.

#### Milk samples

Milk is considered a complete food with a source of protein and major minerals. A total of 125 samples of milk collected which comprises raw milk, pasteurized milk, and UHT milk were screened for pesticide using paper strip-based senor. Among 125 samples of milk comprising 89 raws, 12 pasteurized and 24 UHT milk among that 31 samples of raw milk and 2 samples of pasteurized milk found positive for pesticide (Fig. 2C). The assessment and detection of organochlorine pesticide (OC) residues in bovine milk of different places in the Bundelkhand region of India were studied by Nag and Raikwar^26^. They revealed that the concentration of pesticide levels in milk was decreased over the previous studies of particular in India but still contamination present in low concentration. Overall, 33 milk samples, 6 cereal flour samples, and 4 fruit juice samples were found positive for pesticide residues (Fig. 2D) by developed paper strip sensors for detection of pesticide residues.

#### GC–MS/MS analysis

**Figure 3:**
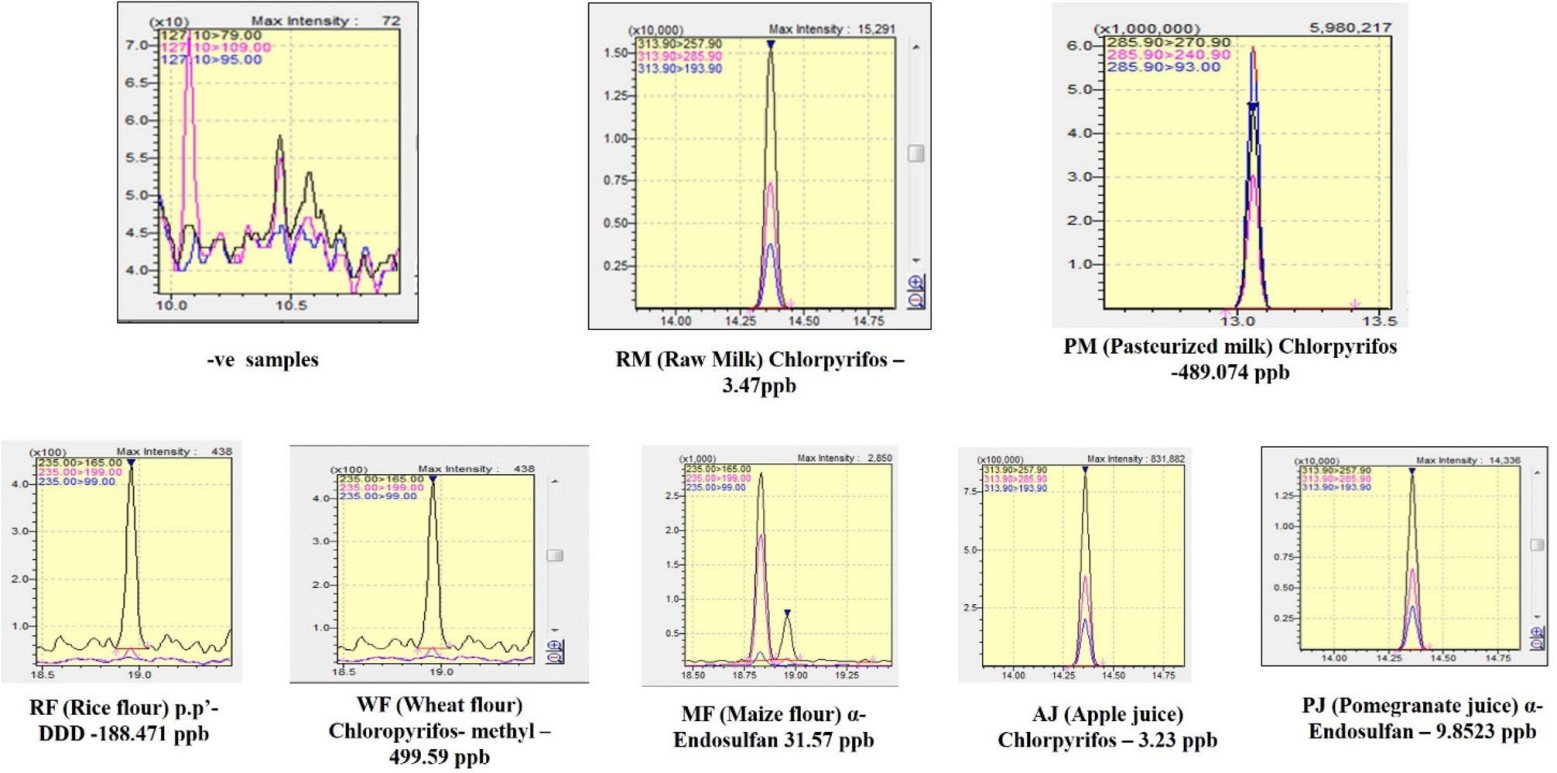
Confirmation of pesticides residues in milk, cereal based foods, and fruit juices quantitatively by GC–MS/MS.

Among 43 positive samples out of 159 samples including milk, cereal products, and fruit juice samples by paper strip sensor for detection of pesticide residues were evaluated for pesticide residues quantitatively by GC–MS/MS. The condition required for multiple reactions monitoring (MRM) method using GC–MS/MS was optimized for the analysis of pesticide residues in food samples (Table 2). In GC–MS/MS analysis about 11 pesticides were targeted from two groups that include organochlorine (Aldrin, dieldrin, endosulfan, and DDT) and organophosphate (Fenithrothion, Chlorpyrifos- methyl, Monochrotofos, Diazinon, Malathion, Phorate, and Chloropyrifos). Among the 43 samples, 7 samples (Raw milk, pasteurized milk, rice flour, wheat flour, maize flour, apple juice, and pomegranate juice) were found positive for different groups of pesticides at trace levels or above the MRL using GC–MS/MS analysis (Fig. 3). Three samples of pasteurized milk, rice flour, and wheat flour were positive for the presence of Chlorpyrifos and chlorpyrifos-methyl pesticide residues, respectively at above MRL level prescribed by the Codex Alimentarius Commission (Table 3). In case of other pesticide residues found at trace level that includes DDT, DDD, α- Endosulfan, β-Endosulfan that are found at the level below MRL using GC–MS/MS and these samples were showing positive by our developed paper strip assay. Based on the above comparative study it can be concluded that the developed paper strip assay can be a potential tool in the screening of pesticide residues in a large number of milk samples, cereal flour samples, and fruit juice samples. Similar results were reported by Kowalska et al.^27^ in the products of plant origin and the pesticides identified by HPLC–MS/MS includes azoxystrobin (22.5%), linuron (20.6%), chlorpyrifos, and carbendazim (8.1%), metalaxyl and metalaxyl M (6.9%), and acetamiprid (4.4%). In India, Dwivedi et al.^28^ have reported the presence of Chlorpyrifos in orange juice (1.08 mg/kg) & Deltamethrin (1.28 mg/kg) in ginger garlic at or above the permissible limit of FSSAI. Garcı´a-Reyes et al.^29^ were also reported the presence of pesticide residues in fruit-based soft drinks extracts based on the application of liquid chromatography-electrospray time of-flight mass spectrometry (LC-TOF MS). The contamination of milk with hexachlorocyclohexane (HCH), dichloro-diphenyl trichloroethane (DDT), endosulfan, cypermethrin, cyhalothrin, permethrin, chlorpyrifos, ethion, and profenophos pesticides was also reported in peri-urban bovine milk at or above respective maximum residue limits (MRLs) for pesticide using GC–MS^30^.

**Table 2:** Optimized conditions of multiple reactions monitoring (MRM) method for analysis of pesticide.

Compound	Start time	End time	Event time	CH-1 (m/z)	CE	CH-2 (m/z)	CE	Q1 Resolution	Q3 Resolution
Monocrotophos	9.86	11.14	0.15	127.10 > 109.00	12	127.10 > 95.00	16	Low	Low
Phorate	9.86	11.14	0.15	260.00 > 75.00	8	260.00 > 231.00	4	Low	Low
Diazinon	11.14	12.35	0.3	304.10 > 179.10	10	304.10 > 162.10	8	Low	Low
Chlorpyrifos-methyl	12.35	14.47	0.06	285.90 > 93.00	22	285.90 > 270.90	14	Low	Low
Fenitrothion	12.35	14.47	0.06	277.00 > 260.00	6	277.00 > 109.10	14	Low	Low
Malathion	12.35	14.47	0.06	173.10 > 99.00	14	173.10 > 127.00	6	Low	Low
Chlorpyrifos	12.35	14.47	0.06	313.90 > 257.90	14	313.90 > 285.90	8	Low	Low
Aldrin	12.35	14.47	0.06	262.90 > 193.00	28	262.90 > 203.00	26	Low	Low
alpha-Endosulfan	15.75	18.32	0.15	338.90 > 160.00	18	338.90 > 266.90	8	Low	Low
Dieldrin	15.75	18.32	0.15	276.90 > 241.00	8	276.90 > 170.00	38	Low	Low
beta-Endosulfan	18.32	19.61	0.15	338.90 > 160.00	18	338.90 > 266.90	8	Low	Low
p,p'-DDD	18.32	19.61	0.15	235.00 > 165.00	24	235.00 > 199.00	14	Low	Low
p,p'-DDT	19.61	20.75	0.3	235.00 > 165.00	24	235.00 > 199.00	16	Low	Low

**Table 3:** Comparative analysis of food samples in Paper strip sensor and GC–MS.

S.No	Sample Code	Results obtained by GC–MS/MS analysis			Results on paper strip
		Pesticide found	Concentration (ppb)	MRL	
1.	RM (Raw milk)	Chloropyrifos	3.47	Below	Positive
		DDT	0.012	Below	Positive
2.	PM (Pasteurized milk)	Chloropyrifos- methyl	489.074	**Above**	Positive
		DDD	0.06702	Below	Positive
3.	RF (Rice flour)	Chloropyrifos	188.471	**Above**	Positive
		DDT	0.01700	Below	Positive
4.	WF (Wheat flour)	Chloropyrifos- methyl	499.59	**Above**	Positive
		DDD	0.0755	Below	Positive
5.	MF (Maize flour)	α- Endosulfan	31.57	Below	Positive
6.	AJ (Apple juice)	Chloropyrifos	3.23	Below	Positive
7.	PJ (Pomegranate juice)	α- Endosulfan	51.8389	Below	Positive
		β—Endosulfan	9.8523	Below	Positive
		DDT	0.0332	Below	Positive

#### Analytical performance

The developed paper strip based sensor was evaluated for its analytical performance in comparison with GC–MS/MS system. The developed sensor has shown 33 true positive samples, and 2 false negative samples in comparison with GC–MS/MS system. The sensor has shown 97.6% accuracy, 0.9428 precision, 97% sensitivity, and 97.8% specificity in comparison with reference method i.e., GC–MS/MS system. Pum^31^ has shown accuracy, precision, true positive rate, false positive rate of qualitative drug abuse screening assay wherein he found 97.5% accuracy, 5.2% accuracy, 100% True positive rate, and 5.6% false positive rate, respectively. Jiang et al.^32^ combined deep learning and machine vision to predict the pesticide. The consequences showed that when the training epoch is 10, the precision of the test set detection will be 90.09% and the average picture bandwidth detection precision will be 95.35%.

## Conclusion

The current study shows that developed extraction protocol gives better detection of pesticides from milk, cereal-based food, and fruit juices. This paper strip-based sensor cannot differentiate the pesticide groups its shows only presence and absence of pesticide in food sample. So, this can help in initial screening of pesticide in large number of food sample. Than the positive samples can further go for GC–MS for confirmation of pesticide group. Hence it can decrease the sample load for GC analysis. Obtained results show a wide range of applications of paper strip sensors and found to be a cost-effective, robust, and rapid method for the detection of pesticides from samples. The comparative result obtained of pesticide-positive samples using GC–MS were ranged between 0.012–499.59 ppb. There are no losses of pesticides during extraction from food samples. Further, analytical performance of the sensor for the screening of milk, cereal based food, fruits juices were found to accurate, better precision, better true positive results, and minimum false negative results. The detection limit of pesticide from the food sample ranges from 1–500 ppb which is below the MRL of pesticide according to FSSAI and EU regulatory standards. Based on the above, it would be potential techniques for screening of large number of food samples under field conditions.
